# Transcriptome Profiling Unveils the Mechanisms of Inflammation, Apoptosis, and Fibrosis in the Liver of Juvenile Largemouth Bass *Micropterus salmoides* Fed High-Starch Diets

**DOI:** 10.3390/ani14233394

**Published:** 2024-11-25

**Authors:** Xifeng Liu, Hongkang Liu, Kangwei Wang, Chuanjie Qin, Yuanfa He, Li Luo, Shimei Lin, Yongjun Chen

**Affiliations:** 1 Integrative Science Center of Germplasm Creation in Western China (CHONGQING) Science City, Key Laboratory of Freshwater Fish Reproduction and Development (Ministry of Education), College of Fisheries, Southwest University, Chongqing 400715, Chinam17634418632@163.com (H.L.);; 2 Key Laboratory of Sichuan Province for Fishes Conservation and Utilization in the Upper Reaches of the Yangtze River, Neijiang Normal University, Neijiang 641100, China; qinchuanjie@126.com

**Keywords:** *M. salmoides*, feed formulation, histology, liver injury, molecular mechanism

## Abstract

The aim of this study was to explain the mechanism underlying the liver injury of juvenile largemouth bass *Micropterus salmoides* in response to high-starch diet intake. Three diets were formulated with different starch levels, being abbreviated as treatment LS (low starch, 8.13% starch), MS (medium starch, 14.1% starch), and HS (high starch, 20.1% starch), respectively. Fish were fed with their respective diets to apparent satiation for 56 days. The results showed that growth retardation of the HS fish was associated with the reduction in feed intake rather than feed utilization. Histological evaluation of the livers showed that vacuolization was the most prevalent characteristic in the MS fish, while ballooning degeneration, apoptosis, fibrosis, and inflammation were observed in the HS fish. Transcriptome profiling suggested that liver inflammation was mediated by Tlr signal transduction, which activated the Pi3k/Akt/Nfκb signaling axis to promote the release of proinflammatory factors including Il-8 and Ip-10. Hepatocyte apoptosis was mediated by the extrinsic pathway through death receptors including Fas and Tnfr, which coordinately activated the Fadd/caspase-8 death signaling axis. An autonomous inhibition program was identified to counteract the apoptosis signal, and the PI3K/Akt signaling pathway might play an important role in this process through regulating the expression of *iap* and *diablo*. Liver fibrosis was mediated through the Tgf-β and Hh signaling pathways. Upon secretion, Tgf-β1/3 bound to TgfβrI/II complex on the liver cell membrane, which induced the phosphorylation of downstream Smad2/3. When Hh interacted with the membrane receptor Ptc, Smo was activated to initiate signaling, driving the activation of Gli. The activation of both Smad2/3 and Gli promoted their nuclear translocation thereby regulating the transcription of target genes, which resulted in the activation and proliferation of HSCs. The activated HSCs constantly expressed *colla1* and *ctgf*, which facilitated substantial accumulation of ECM. It should be noted that the molecular mechanism of liver injury in this study was speculated from the transcriptome data thus further experimental verification is warranted for this speculation.

## Simple Summary

It is well known that the application of high-starch diets induces liver injury in largemouth bass *Micropterus salmoides* (LMB), but the underlying mechanism is still largely unclear. In this study, an 8-week feeding trial was sufficient to induce liver damages such as inflammation, apoptosis and fibrosis in LMB fed diets with 20.1% starch. In line with the histological changes, KEGG enrichment analysis showed that DEGs in the liver of the fish fed high-starch diets were prominently enriched in regulation of actin cytoskeleton, apoptosis, cytokine-cytokine receptor interaction, NOD-like receptor signaling pathways, and so on. Transcriptome analysis revealed that liver inflammation was mediated by the Tlr signal transduction via the Pi3k/Akt/Nfκb signaling axis, apoptosis was mediated by the extrinsic pathway, while fibrosis was mediated through the Tgf-β and Hh signaling pathways. Our results provided new insights into the molecular mechanism underlying the liver injury of LMB fed high-starch diets.

## 1. Introduction

As the main source of energy, carbohydrates are widely present in our daily diet. Digestible carbohydrates are converted into monosaccharides for absorption and metabolized to produce energy [1]. With the improvement of living standards, the prevalence of high-carbohydrate diets in human beings is closely related to the incidence of nonalcoholic fatty liver disease, which is a chronic disease that can progress to steatohepatitis and in severe cases to hepatocellular carcinoma [2]. In mammals, the molecular mechanism and therapeutic targets of metabolic liver diseases resulting from high-carbohydrate diet intake have been extensively investigated [3,4]. Carbohydrate is an indispensable ingredient in aquafeeds not only as the binding agent to facilitate feed pelleting, but also as the least cost energy source to save protein for maximal somatic growth [5,6]. In fish however studies regarding the effects of dietary carbohydrates on liver health are still at the epiphenomenal level due to their huge difference in glucose tolerance with different feeding habits. Although a number of researchers have suggested that high-carbohydrate diet intake could induce excessive lipid deposition, causing a disturbance of intermediary metabolism or pathological changes in the liver of fish [7,8,9], the underlying regulatory mechanisms are still largely unclear.

Largemouth bass *Micropterus salmoides* (LMB) has been favored by farmers and the market in China since 1983 when it was introduced into Guangdong province [10]. The culture scale and production of LMB have been increasing especially in the last decade, making it one of the most important freshwater fish in China. The capability of LMB to utilize dietary carbohydrates is lower than that of other carnivorous fish. The upper limit of dietary digestible carbohydrate (starch) for most carnivorous fish is 20% [11], while this value for LMB is less than 10% [12]. Currently, it is still not certain whether fatty liver symptoms could be induced by high-starch diet intake (>10%) in LMB, as contradictory results were reported regarding whether excessive starch in the diet could be converted into lipid for storage [13,14]. However, it is undoubted that feeding high-starch diets could induce signs of liver injuries including inflammation, apoptosis, and/or fibrosis in LMB [15]. In recent years, several researchers have employed omics approaches to unveil the underlying mechanism of liver injury in LMB fed high-starch diets [13,16], but the results differed with each other possibly due to the differences in dietary starch levels and experimental duration. Zhong et al. [15] reported that the Pi3k/Akt signaling pathway probably mediated the development of liver fibrosis in LMB fed diets with 22% starch, but the downstream transcriptional factor is not yet determined. However, our previous study suggested that the Pi3k/Akt/Nfκb signaling pathway played an important role in liver inflammation and apoptosis of LMB fed low-protein, high-starch diets [17]. In order to exclude the potential effects of dietary protein, only the dietary starch level was changed in this study to evaluate its impact on growth performance, nutrient composition, and liver pathology and transcriptome of LMB. The results of this study are helpful to elucidate the role of the Pi3k/Akt signaling pathway in liver injury of LMB fed high-carbohydrate diets.

## 2. Materials and Methods

### 2.1. Experimental Diets

The formulation and proximate composition of the diets are presented in Table 1. Three isonitrogenous (i.e., 49.4% protein) and iso-lipidic (i.e., 11.5%) diets were prepared with different starch levels (8%, 14%, and 20%), being designated as treatments LS (low starch as the control), MS (medium starch), and HS (high starch), respectively. Cassava starch was used as the carbohydrate source at the expense of microcrystalline cellulose and zeolite powder. Dietary cellulose level was maintained below 7% according to Zhong et al. [15]. Due to the use of non-fish meal protein sources, such as wheat gluten, chicken meal, and soybean meal, crystalline methionine was supplemented in the diets to meet the minimum requirement of LMB juveniles [7]. The raw feed ingredients were completely crushed and fully mixed in proportion. Then, the diets were prepared by a feed expander. The feed pellets were air-dried, sealed in valve bags, and stored at −20 °C in a freezer until fed.

### 2.2. Experimental Fish and Feeding Management

About 400 juvenile LMB (about 3 g per fish) were purchased from Chongqing Cross-Strait Agricultural Development Co., Ltd. (Beibei, Chongqing, China). After transient disinfection with 3% saline, the fish were kept in our facility for 15 days. During this period, they were fed with a commercial diet (Guangdong Haida Group Co., Ltd., Guangzhou, China) twice a day to near satiation.

After the acclimatization, 180 LMB juveniles of average weight 6.37 ± 0.07 g were selected and transferred into 9 rectangular glass tanks (200 L each, 20 fish per tank) operating as a recirculating aquaculture system (RAS). Three replicate tanks were set up for each diet. During the next 56 days, the fish were fed their respective diet manually twice daily (09:00 and 17:00). Each tank of fish was bulk-weighed and counted every 4 weeks to evaluate the growth performance and survival. During the feeding trial, the water temperature fluctuated between 26.3 and 29.9 °C, and the other water quality parameters (pH, dissolved oxygen ammonia nitrogen, and nitrite) were well maintained.

### 2.3. Sampling

After the end of the feeding, 13 fish were randomly taken from each tank after 24 h of fasting. Among which 3 fish were used for whole body composition analysis, and 6 fish were used for blood collection from the caudal vein using a sterile syringe. The blood was kept at 4 °C overnight and then centrifuged to obtain serum by stratifying the blood. The serum samples were stored at −20 °C in a freezer pending further biochemical analysis. Prior to blood collection, the body weight and body length of each fish were measured. After the blood collection, each fish was dissected, and viscus, liver, and intraperitoneal fat were separated and weighed to calculate morphological indicators. The livers were collected and pooled per tank for proximate analysis. The remaining four fish were dissected to obtain the liver samples. The proper amount of liver pieces were fixed in 4% paraformaldehyde fixative for histological analysis. The remaining livers were immediately frozen in liquid nitrogen and then transferred to −80 °C in an ultra-low temperature refrigerator for real-time PCR analysis and transcriptome sequencing.

### 2.4. Nutrient Composition Analysis

Moisture, crude lipid, crude protein, and crude ash were determined according to standard procedures [18]. Diet samples were sent to Guangzhou Ashare Aquatech Co., Ltd. for determining starch levels according to the method of Hall [19]. Glycogen concentration of the liver was determined according to the instructions of a commercial kit (Cat. No. A043-1-1) from the Nanjing Jiancheng Institute of Biological Engineering, Nanjing, China.

### 2.5. Serum Biochemical Analysis

Total protein, triglyceride, and cholesterol levels were determined using commercial kits (Nanjing Jiancheng Institute of Biological Engineering) with the Cat. No. of A045-2, A110-2-1, and A111-2-1, respectively.

### 2.6. Histological Examination

The liver pieces were entrusted to Wuhan Servicebio Technology Co., Ltd. (Wuhan, China) for paraffin embedding, sectioning, hematoxylin-eosin (HE) staining, and Masson staining. The TUNEL (terminal deoxynucleotidyl transferase-mediated nick end labeling) method was used to detect DNA fragmentation of apoptotic hepatocytes according to the instructions of a commercial kit (C1088, Biyuntian, Shanghai, China). The nuclei were stained with 4,6-diamidino-2-phenylindole (DAPI). The images were captured using the Olympus DP73 optical microscope or Olympus FV3000 fluorescence microscope (Tokyo, Japan) as required. The green fluorescence intensity of TUNEL positive staining was quantified with ImageJ 1.43 per 1000 μm^2^ area.

### 2.7. Transcriptome Analysis

#### 2.7.1. cDNA Library Construction, Sequencing, and Transcriptome Assembly

Liver samples of the LS and HS fish were transported in dry ice and entrusted to Biomarker Technologies Co., Ltd., (Beijing, China) for next-generation sequencing. Total RNA extraction, reverse transcription, cDNA library generation, and sequencing on the Illumina platform were performed according to Shen et al. [20]. Adaptor containing, low quality sequences, and ploy-N were removed from the raw reads. Sequence repeat levels and Q30 and GC content of the clean reads were then calculated. Transcriptome assembly was fulfilled according to standard procedures as described by Grabherr et al. [21].

#### 2.7.2. Differential Expression Analysis

Differential expression analysis was performed based on the Wald test with DESeq2. DESeq2 fitted a model with a negative binomial distribution to determine the differential expression of numerical gene expression. Differential gene expression was calculated using count value analysis as follows: sequencing the sample to obtain the reads value, comparing with the reference genome, obtaining the counts value, and normalizing the counts. The false discovery rate was examined using Benjamini and Hochberg’s method to reconcile the obtained *p* values. Differentially expressed genes (DEGs) were screened with the standard of FC (fold change) >1.5 and *p* < 0.05.

#### 2.7.3. Functional Annotation and Enrichment Analysis

Functional annotation of the DEGs was carried out against the gene ontology (GO) and Kyoto Encyclopedia of Genes and Genomes (KEGG) databases. GO enrichment of the DEGs was performed using the topGO R program package (version 2.58.0.) with the Kolmogorov–Smirnov test [22]. KEGG enrichment of the DEGs was performed using the R clusterProfiler program package (version 3.20). The degree of pathway enrichment and significance of enrichment were calculated with enrichment factors and Fisher’s exact test, respectively.

### 2.8. Real-Time PCR Analysis

The reliability of transcriptome sequencing was verified by quantitative real-time PCR. Primer sets for target genes were designed by Primer Premier 5 (Table 2). Total RNA extraction, RNA quality and concentration assessments, cDNA preparation, and real-time PCR analysis were prepared according to the procedures of our previous research [23]. Before real-time PCR analysis, regular PCR amplifications were carried out to verify the specificity of the primer sets and the size of the PCR products on a 1.5% agarose gel. The real-time PCR amplifications were performed in 20 μL reactions with a mixture of 0.8 μL of each 10 μM primer, 2 μL cDNA, 10 μL 2 × SYBR Premix ExTaq (TaKaRa, Shiga, Japan), and 6.4 μL of PCR-grade water. The PCR reactions were initiated by initial denaturation at 95 °C for 1 min, followed by 39 cycles at 95 °C for 5 s, 60 °C for 30 s, and 72 °C for 30 s, and then 95 °C for 15 s, 60 °C for 60 s, and 95 °C for 15 s to obtain the melt curve. The amplification efficiency was then calculated.

### 2.9. Calculations

Feed efficiency ratio = (final body weight-initial body weight)/feed intake;

Protein efficiency ratio = (final body weight-initial body weight)/protein intake;

Feeding ratio (%) = 100 × feed intake × 2/(final body weight + initial body weight)/experimental duration in days;

Condition factor (%) = body weight/body length^3^ × 100;

Viscerosomatic index (%) = (viscus weight/body weight) × 100;

Hepatosomatic index (%) = (liver weight/body weight) × 100;

Intraperitoneal fat ratio (%) = (intraperitoneal fat weight/body weight) × 100.

### 2.10. Statistical Analysis

Results are expressed as mean ± SD (standard deviation). Data for real-time PCR analysis and TUNEL positive fluorescence intensity between the LS and HS fish were compared by an independent sample T test. The remaining data were analyzed using one-way analysis of variance (ANOVA) and Tukey’s multiple tests to identify the significance among three treatments. The minim significant level was set at 0.05. Before one-way ANOVA, the data were first validated for the normality of distribution (one-sample Kolmogorov–Smirnov test) and homogeneity of variances (Levene’s test). All the statistical analysis was performed using SPSS 22.0 (SPSS, Chicago, IL, USA).

## 3. Results

### 3.1. Growth, Feed Utilization, and Morphological Parameters

**Figure 1:**
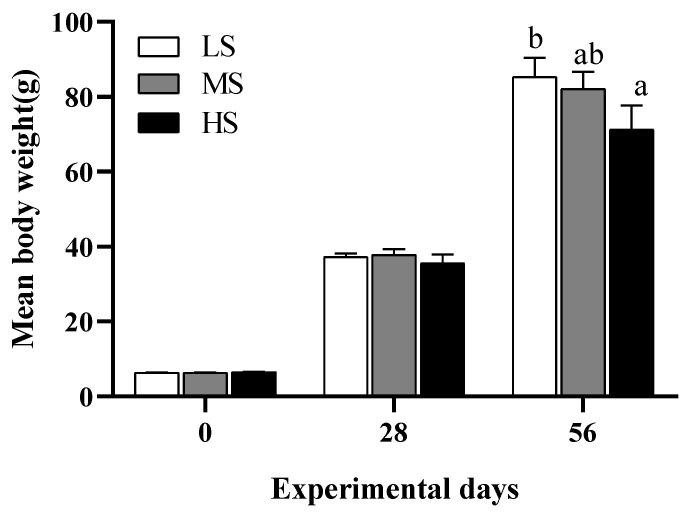
The growth of largemouth bass *M. salmoides* fed different starch levels during the feeding trial. Different letters on the error bars indicate significant differences among treatments (*p* < 0.05). LS, low starch; MS, medium starch; HS, high starch.

Changes in mean body weight of LMB during the feeding trial are shown in Figure 1. After 4 weeks of the feeding trial, the mean body weight of LMB (35.0~37.8 g) was not significantly differentiated among the three treatments (*p* > 0.05). However, the final mean body weight was gradually decreased with increasing dietary starch levels at the end of the 8th week, and the difference between the LS (85.3 g) and HS fish (71.3 g) was significant (*p* < 0.05). Data for feed utilization and morphological parameters of LMB are shown in Table 3. Both feed efficiency ratio and protein efficiency ratio were gradually increased with increasing dietary starch levels, and there were significant differences between the LS and HS fish (*p* < 0.05). Feeding ratio followed an opposite trend to feed efficiency ratio and protein efficiency ratio. Viscerosomatic index (VSI) of the LS fish (8.31%) was significantly lower than those of the MS (9.81%) and HS fish (9.85%; *p* < 0.05). Hepatosomatic index (HSI) was significantly increased with elevated dietary starch levels from 8% to 20% (*p* < 0.05). Condition factor and intraperitoneal fat ratio (IPF) were not affected by dietary starch levels (*p* > 0.05).

### 3.2. Serum Biochemical Indices

Blood glucose and serum biochemical indices of LMB are shown in Table 4. Dietary starch levels did not significantly affect fasting blood glucose (2.60~3.04 mmol/L; *p* > 0.05). Triglyceride (11.1~13.3 mmol/L), cholesterol (10.8~12.0 mmol/L), and protein (21.1~24.0 g/L) levels in the serum were also not significantly differentiated among the three treatments (*p* > 0.05).

### 3.3. Nutrient Composition

Nutrient composition in the whole body and liver of LMB are shown in Table 5. Proximate composition (moisture, protein, lipid, and levels) of the whole fish were not affected by dietary starch levels (*p* > 0.05). Protein level in the liver of the LS fish (11.2%) was significantly higher than those of the MS (8.55%) and HS fish (7.83%; *p* < 0.05). However, liver moisture and lipid levels were not significantly affected by the dietary starch levels (*p* > 0.05). Liver glycogen concentration increased significantly when the dietary starch level increased from 8% to 20% (*p* < 0.05).

### 3.4. Histological Observation

**Figure 2:**
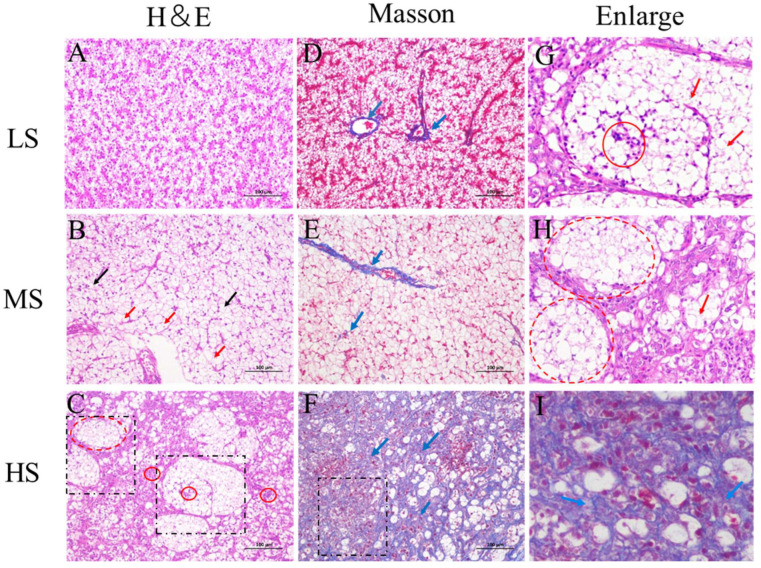
HE and Masson staining of the liver sections in largemouth bass *M. salmoides* fed different starch levels. LS, low starch; MS, medium starch; HS, high starch. (**A**–**C**) are HE staining of livers from the LS, MS, and HS fish, respectively. (**D**–**F**) are Masson staining of liver sections from the LS, MS, and HS fish, respectively. (**G**–**I**) are the magnification of the black dashed rectangular region in (**C**,**F**). The red arrow indicates vacuolation. The black arrow indicates nuclear migration. The red circle indicates inflammatory infiltration. The red dashed circle indicates ballooning degeneration. The blue arrow indicates fibrous scars.

**Figure 3:**
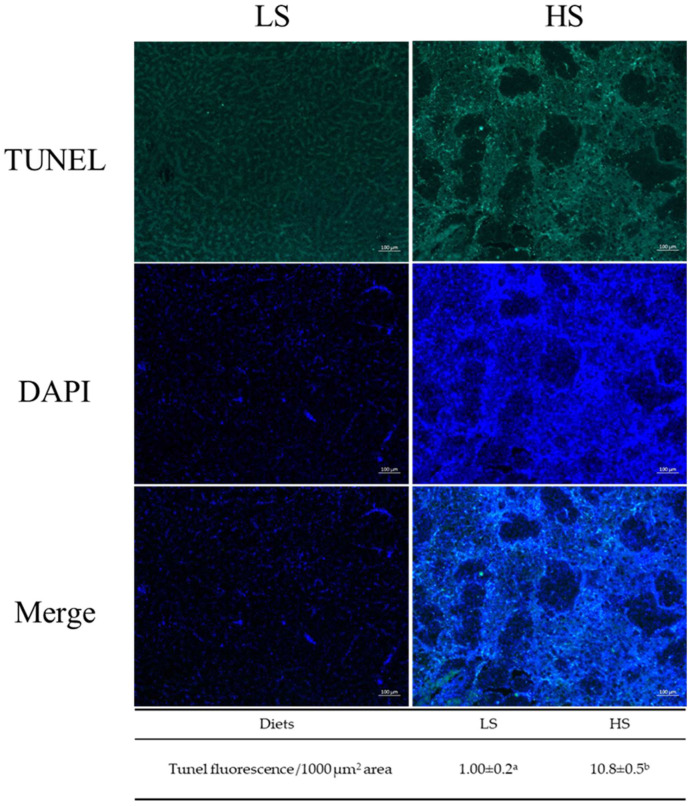
Effects of dietary starch levels on hepatocyte apoptosis of largemouth bass *M. salmoides* fed different starch levels. Green indicates TUNEL positive staining. Blue indicates DAPI nuclear staining. Different letters in same row indicate significant differences among treatments (*p* < 0.05). LS, low starch; HS, high starch.

Histological characteristics for HE and Masson staining of the liver sections are shown in Figure 2. HE staining showed that hepatocytes were intact, hepatic cords of the LS fish were arranged neatly, and the nucleus was centered. The prominent characteristic of liver sections in the MS fish was severe vacuolization (indicated by the red arrow) with deviated or even disappeared nuclei, while ballooning degeneration (the red dashed circle) with inflammatory infiltration (the red circle) was identified as the most characteristic of the HS fish. Masson staining showed that fibers (the blue arrow) were only distributed around the blood vessels in the liver of the LS fish, while several intercellular fibers were observed around the hepatocytes in the MS fish. In the HS fish, fibers were widespread around the hepatocytes. As shown in Figure 3, TUNEL positive green fluorescence intensity in the liver sections was 10.8-fold higher in the HS fish than in the LS fish (*p* < 0.05), suggesting that hepatocyte apoptosis was induced in LMB fed with increasing dietary starch levels from 8% to 20%.

### 3.5. Quality Assessment and Sequence Alignment of Transcriptome

In this study, six cDNA libraries were constructed from the livers of the LS and HS fish. After removing the low-quality sequence, a total of 36.62 Gb clean data were obtained with at least 93.9% of Q30 bases per sample (Table S1). The GC content of the cDNA libraries ranged from 47.7% to 48.6%. Sequence alignment analysis showed that 94.2%~95.0% of the total reads could be mapped to the LMB reference genome (Table S2).

### 3.6. Differential Expression Analysis

**Figure 4:**
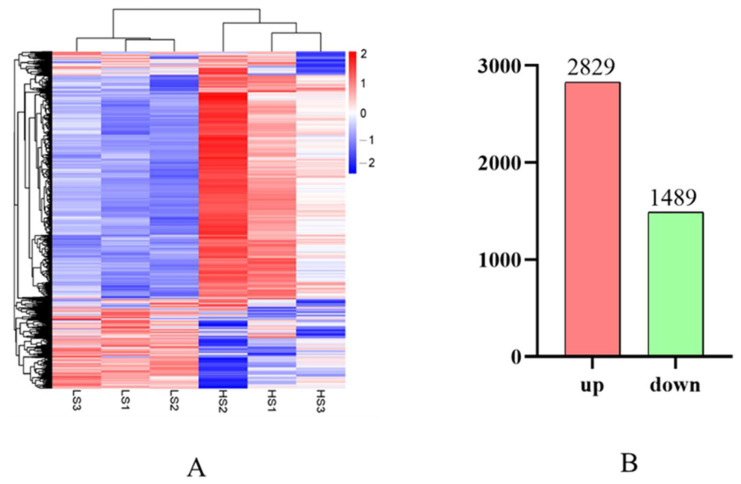
Clustering heat map and differential expression analysis of transcriptome data. (**A**) Heat map generated by hierarchical clustering of DEGs. Blue, white, and red gradient shading indicate low, medium, and high gene expression levels, respectively. (**B**) Red and green columns indicate numbers of up- and down-regulated DEGs, respectively.

A clustering heat map showed that DEGs from the same treatment clustered together (Figure 4A). Compared with the LS fish, a total of 4417 DEGs were screened out in the liver transcriptome of the HS fish. Among which 2928 and 1489 DEGs were up- and down-regulated, respectively (Figure 4B).

### 3.7. GO and KEGG Enrichment Analysis

**Figure 5:**
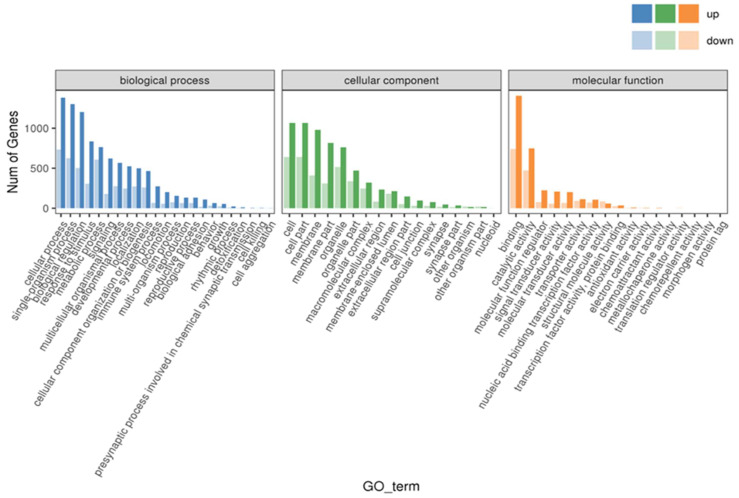
The GO enrichment of the DEGs from the livers of largemouth bass *M. salmoides* fed high-starch diets as compared to the fish fed low-starch diets.

As shown in Figure 5, GO enrichment analysis classified the DEGs into three categories: biological processes (BP), cellular components (CC), and molecular functions (MF). Compared with the LS fish, the top three enriched subclasses of the DEGs in the HS fish were: cellular process, single-organism process, and biological regulation in the BP; cell, cell part, and membrane in the CC; and binding, catalytic activity, and molecular function regulator in the MF.

**Figure 6:**
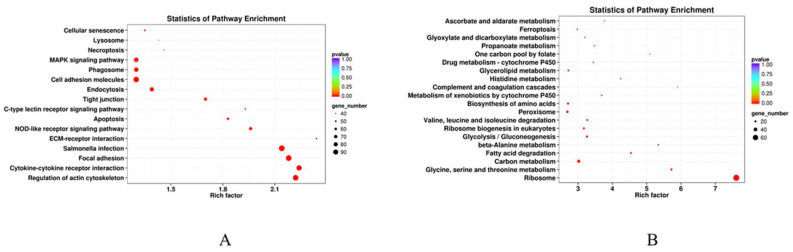
KEGG enrichment of DEGs from livers of largemouth bass *M. salmoides* fed high-starch diets as compared to fish fed low-starch diets. (**A**,**B**) represent enrichment analysis of up- and down-regulated DEGs, respectively.

Aa shown in Figure 6A, KEGG enrichment analysis showed that the up-regulated DEGs in the liver of the HS fish were prominently enriched in salmonella infection, focal adhesion, regulation of actin cytoskeleton, cell adhesion molecules, and cytokine–cytokine receptor interaction. In addition, MAPK signaling pathway, NOD-like receptor signaling pathway, apoptosis, and ECM–receptor interaction were also significantly enriched (Table S3). Compared with the LS fish, the down-regulated DEGs in the liver of the HS fish were prominently enriched in ribosome and carbon metabolism (Figure 6B).

### 3.8. Real-Time PCR Analysis

**Figure 7:**
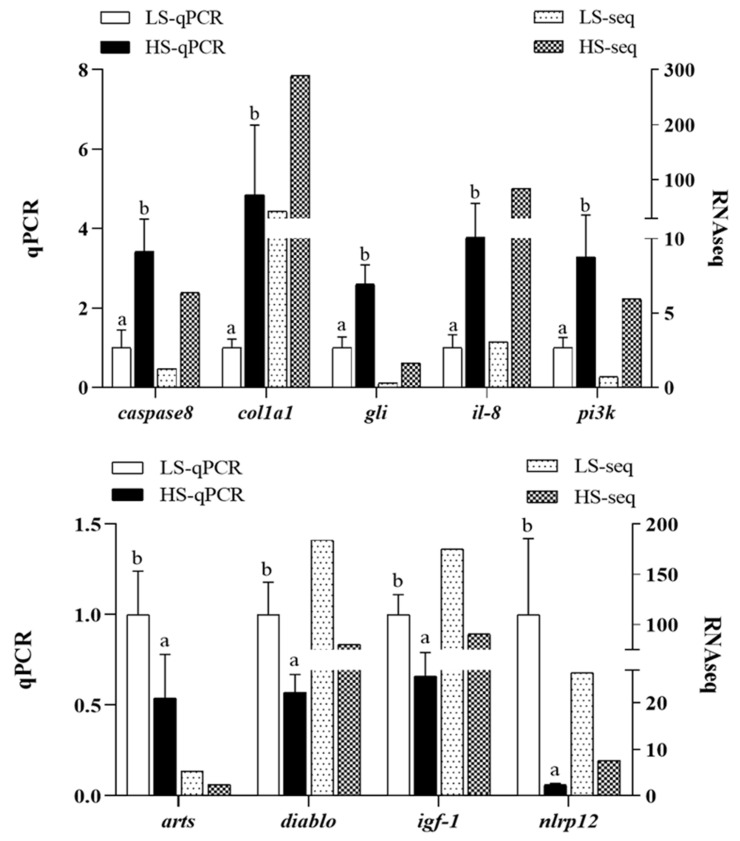
Validation of normalized count value of DEGs by real-time PCR analysis. Different letters on error bars indicate significant differences among treatments (*p* < 0.05). LS, low starch; HS, high starch.

In order to evaluate the reliability of the transcriptome data, real-time PCR analysis was performed to quantify the mRNA levels of nine target genes. Consistent with the transcriptome data, the mRNA levels of *caspase 8*, *col1a1*, *gli*, *il-8*, and *pi3k* were significantly up-regulated, while the expression of the remaining target genes was significantly down-regulated in the liver of the HS fish compared with the LS fish (*p* < 0.05; Figure 7).

### 3.9. Putative Molecular Mechanism of Liver Injury

**Figure 8:**
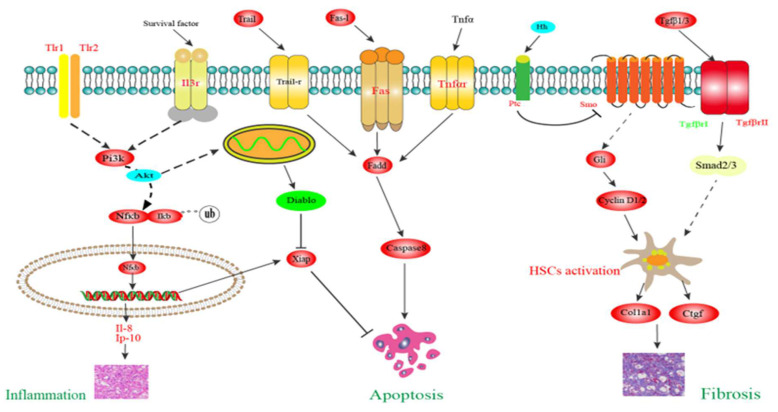
Putative mechanism underlying liver inflammation, apoptosis, and fibrosis of largemouth bass *M. salmoides* fed high-starch diets. Red indicates up-regulated, blue indicates both up- and down-regulated, while green indicates down-regulated DEGs. Akt, protein kinase B; Col1a1, type I collagen; caspase 8, cysteinyl aspartate specific proteinase 8; Ctgf, connective tissue growth factor; diablo, direct IAP binding protein with low pI; Fadd, Fas-associating protein with a novel death domain; Fas, factor associated suicide; Fas-l, factor associated suicide ligand; Gli, glioma-associated oncogene homolog; Hh, hedgehog; HSCs, hepatic stellate cells; Iκb, inhibitors of Nfκb; Il-8, interleukin-8; Ip-10, interferon inducible protein 10; Nfκb, nuclear factor kappa-B; Pi3k, phosphatidylinositol 3-kinase; Ptc, patched homolog; Smo, smoothened homolog; Tgf, transforming growth factor; Tgfβr, transforming growth factor β receptor; Tlr, toll-like receptor; Tnf, tumor necrosis factor; Tnfr, tumor necrosis factor receptor; Trail, Tnf-related apoptosis-inducing ligand; Trail-r, Tnf-related apoptosis-inducing ligand receptor; Xiap, X-linked inhibitor of apoptosis protein.

The putative molecular mechanisms of liver inflammation, apoptosis, and fibrosis in the HS fish are shown in Figure 8. In this study, transcriptome data showed that liver inflammation was mediated through the Tlr signaling transduction. The stimulation of both Tlr1 and Tlr2 activated the Pi3k/Akt signaling axis, resulting in the phosphorylation of Iκb and its degradation by ubiquitination. Subsequently, Nfκb was released and entered into the nucleus to upregulate the transcription of proinflammatory cytokines including *il-8* and *ip-10* (Figure S1). Hepatocyte apoptosis was mediated by extrinsic pathway through the death receptors including Fas, Tnfr, and Trail-r, which coordinately activated the Fadd/caspase-8 death signaling axis (Figure S2). An autonomous inhibition program was identified to counteract the apoptosis signal, and the PI3K/Akt signaling pathway played an important role in this process through regulating the expression of *iap* and *diablo*. Liver fibrosis was mainly mediated through the Tgf-β and Hh signaling pathways. Upon secretion, Tgf-β1/3 bound to TgfβrI/II complex on the liver cell membrane, which induced the phosphorylation of downstream Smad2/3. When the Hh ligand interacted with the membrane receptor Ptc, Smo was activated to initiate signaling, driving the activation of Gli. The activation of both Smad2/3 and Gli promoted their nuclear translocation thereby regulating the transcription of target genes such as *cyclin D 1/2*, which resulted in the activation and proliferation of HSCs. The activated HSCs constantly expressed *colla1* and *ctgf*, which facilitated substantial accumulation of ECM (Figures S3–S5).

## 4. Discussion

In the present study, an 8 week feeding trial was sufficient to induce growth retardation in LMB fed with 20% starch as compared with fish fed 8% starch. The retarded growth of the HS fish could be a result of the reduction in feeding ratio rather than feed utilization (feed efficiency ratio and protein efficiency ratio). The reason for the reduced feed intake could be attributed to the fact that the energy density of diet HS exceeded the energy requirement of LMB. Consistent with our results, inferior appetite was also associated with high-starch diet intake (15%~22%) in the same species [25,26]. Similarly to the results of Gao et al. [27], higher values of feed utilization indicators were also observed in LMB fed 20% starch than their counterparts fed 8% starch in this study, which should be attributed to the reduced feed intake.

In line with previous findings [26], the VSI of LMB was increased with elevated starch levels from 8% to 14% or 20% in this study. The increase in HSI rather than IPF could be the main reason for the larger viscera size of the HS fish. Consistent with our results, the positive relationship between dietary starch level and liver size was observed in the same species [16] and many other fish species [10,11,28,29]. In this study, the increase in liver size in the HS fish could mainly be a result of the deposition of glycogen accumulation because glycogen deposits are extensively hydrated [30].

In this study, the fasting blood glucose of LMB was not affected by dietary starch levels. The reason might be related to the sampling time, as the peak of postprandial blood glucose in LMB was recorded during 3~12 h, especially at 6 h [25]. In this study, serum triglyceride and cholesterol levels were not differentiated among fish fed different starch levels from 8% to 20%. In addition, lipid levels in the whole fish and liver were also not impacted by dietary starch levels. However, our previous study suggested that triglyceride and cholesterol levels in the serum together with lipid levels in the whole fish and liver of LMB were increased with elevated dietary starch levels from 9% to 14% [17]. It was noteworthy to mention that the rearing condition, feed formulation, and initial body weight of LMB juveniles were similar between these two feeding trials except the genetic background of the fish. The “Youlu 3” strain of LMB was used in our previous study [17]. In this study, we tried to purchase the “Youlu 3” strain from the same producer as well but failed as a result of a severe disease attack. The exact genetic background of LMB in this study was not clear as they were provided by a small-scale local producer. Despite that most of the studies suggested that dietary starch in excess could facilitate lipid deposition in the liver of LMB [15,16,31], Li et al. [26] reported that lipid level was rather decreased in the liver of LMB fed elevated starch from 5% to 15%. It is warranted that the exact genetic background should be provided in future studies as it might impact the hepatic intermediary metabolism of LMB.

The liver plays a pivotal role in important physiological functions such as nutrient absorption, intermediary metabolism, and detoxification in animals including fish. Thus, maintaining liver health is one of the most important issues during the life activities of fish [32]. Starch level in the feed is an important external factor regulating the liver health of LMB. Wu et al. [19] reported that liver fibrosis and inflammation were induced in LMB fed diets containing 28% starch for 4 weeks. Zhong et al. [18] reported that liver fibrosis and hepatocyte apoptosis were induced in LMB fed with 22% starch for 45 days. After an 8 week feeding trial, histological evaluation showed that vacuolization was the most characteristic in the liver of the MS fish, while ballooning degeneration, apoptosis, fibrosis, and inflammation were identified in the liver of the HS fish in this study. It seemed that the characteristics of liver injury in LMB were dependent on dietary starch levels and the experimental duration. To further elucidate the mechanism underlying the liver inflammation, apoptosis, and fibrosis, liver samples of the LS and HS fish were subject to transcriptome sequencing in this study.

A healthy liver stores a large number of immune cells, which are activated to fight against the inflammatory response when liver injury is induced. The failure to clear the inflammatory response can lead to the disruption of liver homeostasis [33]. In line with our previous study [20], transcriptome analysis showed that the Pi3k/Akt/Nfκb signaling pathway still participated in mediating inflammation in the liver of the HS fish in this study. Moreover, the Tlr signal transduction (*tlr1/3*, up-regulated) was identified as the upstream regulator to activate the Pi3k/Akt signaling axis [34], which phosphorylated Iκb and released Nfκb to nuclear translocation thereby promoting the transcription of inflammatory factors including *il-8* and *ip-10* [35,36]. At the same time, the livers of the HS fish initiated chemotactic effects through T cell stimulation (*cd40*, upregulated) and natural killer cells (mip-1β, upregulated) to counteract the inflammatory response [37].

Apoptosis refers to the autonomous and orderly death of cells controlled by genes to maintain the stability of the internal environment. Apoptosis is an active process that is activated by either an exogenous death receptor-mediated pathway or endogenous mitochondrial-initiated pathway [38]. In this study, the apoptosis pathway was enriched by KEGG enrichment analysis. Compared with the LS fish, the transcript levels of *trail*, *fas-l*, *fas*, and *tnfr* were up-regulated in the liver of HS fish. These results suggested that hepatocyte apoptosis of the HS fish was mediated by the extrinsic pathway through the death receptors such as Fas and Tnfr, which coordinately activated the Fadd/caspase-8 death signaling axis [38,39]. The extrinsic and intrinsic apoptosis pathways are not fully independent, as caspase 8 can interact with Bid (up-regulated) for the activation of Bax (up-regulated), which promotes the release of Cyt c from mitochondria [38,40]. In this study, an autonomous inhibition program was identified to counteract the apoptosis signal in the liver of the HS fish, and the Pi3k/Akt signaling pathway might play an important role in this process. On the one hand, the Pi3k/Akt/Nfκb signaling axis can regulate the transcription of *xiap* (up-regulated), which is the most potent apoptosis suppressor in the Iap family and directly inhibits the expression of caspases [41]. On the other hand, Pi3k/Akt reduces the inhibition of Bad on Bcl-2 thereby inhibiting the release of diablo (down-regulated) from mitochondria, which binds Iap to inhibit its anti-apoptotic effect [42,43]. In this study, the reason that the expression of *cyt c* was not changed in the liver of the HS fish might be attributed to the autonomous apoptosis inhibition as mentioned above.

Fibrosis is an uncontrolled wound healing response characterized by the abnormal accumulation of ECM proteins and the formation of fibrous scars [44,45]. HSCs are the main cellular source of ECM protein-secreting-associated myofibroblasts, and their activation is a major driver of liver fibrosis [46]. In this study, several important signaling pathways (focal adhesion, regulation of actin cytoskeleton, cytokine–cytokine receptor interaction, and ECM–receptor interaction) that participated in fibrogenesis were enriched by KEGG enrichment analysis. Among which Tgfβ signal transduction was identified as the upstream regulator in the development of liver fibrogenesis in the HS fish. Upon secretion, Tgfβ1/3 (up-regulated) bound with their membrane receptors TgfβrI and TgfβrII (up-regulated), which subsequently phosphorylated Smad 2/3. The phosphorylated Smad 2/3 formed a polymer and transferred into the nucleus to regulate the transcription of a number of target genes, which could promote the activation and proliferation of HSCs [46,47]. Besides the TGFβ signaling pathway, Hh signal transduction was also identified to play an important role in liver fibrosis of the HS fish. The Hh ligand binding to Ptc (up-regulated)t relieved the inhibitory effect of Ptc on Smo (up-regulated), which abrogated the phosphorylation and degradation of Gli (up-regulated). Gli translocated to the nucleus where it drove the transcription of target genes including *cyclin D1/2* (up-regulated), which promoted the activation and proliferation of HSCs [48,49,50]. Acting as the downstream of Tgfβ signaling, *colla1* and *ctgf* (up-regulated) were constantly expressed in activated HSCs of the HS fish, which promoted substantial ECM accumulation and finally resulted in fibrosis [51,52].

## 5. Conclusions

After an 8 week feeding trial, growth retardation and liver damage were induced in LMB juveniles fed diets with 20% starch. Transcriptome profiling showed that liver inflammation was mediated through Tlr signal transduction via the Pi3k/Akt/Nfκb signaling axis. Hepatocyte apoptosis was mediated by the extrinsic pathway through the death receptors including Fas and Tnfr. An autonomous inhibition program was identified to counteract the apoptosis signal, and the PI3K/Akt signaling pathway might play an indispensable role in this process. Liver fibrosis was mediated through the Tgf-β and Hh signaling pathways. The results of our study provided new insights into the molecular mechanism of liver injury in LMB fed high-starch diets, but investigations at the protein level could be warranted in future studies.

## Figures and Tables

**Table 1:** Formulation and proximate composition of experimental diets (%, dry matter basis).

Ingredients (%)	LS	MS	HS
Fish meal ^1^	34.0	34.0	34.0
Soybean meal ^1^	7.50	7.50	7.50
Wheat gluten ^1^	15.0	15.0	15.0
Chicken meal ^1^	13.0	13.0	13.0
Cassava starch ^1^	7.00	13.0	19.0
Fish oil ^2^	2.40	2.40	2.40
Soybean oil	2.40	2.40	2.40
Monocalcium phosphate ^1^	2.00	2.00	2.00
Vitamin and mineral premix ^3^	2.00	2.00	2.00
Microcrystalline cellulose	6.85	6.00	1.69
Vitamin C phosphate (35%) ^1^	0.20	0.20	0.20
Methionine ^1^	0.25	0.25	0.25
Choline chloride ^1^	0.50	0.50	0.50
Zeolite powder ^1^	6.84	1.69	0.00
Antiseptic ^1^	0.01	0.01	0.01
Antioxidant ^1^	0.05	0.05	0.05
Proximate composition (analyzed, % dry matter)			
Moisture	7.93	9.85	9.64
Crude protein	49.1	49.3	49.8
Crude lipid	11.4	11.6	11.6
Starch	8.13	14.1	20.1
Ash	18.3	13.6	12.0

^1^ Purchased from Chongqing Sitejia Biotechnology Co., Ltd. (Chongqing, China) ^2^ supplied by Chongqing HAID Feed Co., Ltd. (Chongqing, China); ^3^ supplied by Guangzhou Ashare Aquatech Co., Ltd. (Guangzhou, China)

**Table 2:** Primers for real-time PCR of DEGs.

Genes	Forward Primer	Reverse Primer	Product Length (bp)	Tm (°C)	Amplification Efficiency (%)	Accession No.
*atrs*	CCTCTATTTCATCTCACCCTTCG	TCCCATACTGCTCAATCTCCTCT	176	59.8	97.1	XM_038694050.1
*caspase 8*	GAGTAGGGATGAAGTAAAGGC	CTGTAAGGAGAAATGAGGCT	231	52.0	101	XM_038718639.1
*col1a1*	GAGCGGCGAGTATTGGATTG	TGGACATGAGACGCAGGAAAG	269	60.6	101	XM_038692267.1
*diablo*	CCAGACCACCTTGGCTGTCATAG	CTGCTCGGCTCCTGAAGTGTATG	265	59.1	98.1	XM_038723934.1
*gli*	CCGACGGTCCTCTATGGTGT	TGTATTGCTGAGCGGGTGTT	221	58.9	101	XM_038696203.1
*igf-1*	TCTCCTGTAGCCACACCCTCT	GCCTCTATCTCCACACACAAACT	134	57.8	102	XM_038738328.1
*il-8*	TCCTGGCTGCTCTGGCTCTC	GGATGGCCCTCCTGTTAATGG	111	63.1	98.5	XM_038704089.1
*nlrp12*	AAGGAAAGGAGAGGATGACGAC	AAACCCGAACGCAGGATAGA	268	59.1	99.3	XM_038692024.1
*pi3k*	TCTCAAGGGAGGAGGTCA	CCGAATGTCAGAGGGTC	184	55.1	99.3	XM_038730665.1
*eef1a1*	GTTGCTGCTGGTGTTGGTGAG	GAAACGCTTCTGGCTGTAAGG	156	60.1	100	XM_038695351

*atrs*, septin 4b transcript variant x1; *caspase 8*, cysteinyl aspartate specific proteinase 8; *col1a1*, type I collagen; *diablo*, direct iap binding protein with low pi; *gli*, glioma-associated oncogene homolog; *igf-1*, insulin-like growth factors −1; *il-8*, interleukin-8; *nlrp12*, nlr family, pyrin domain containing 12; *pi3k*, phosphatidylinositol 3-kinase; *eef1a1*, eukaryotic translation elongation factor 1 alpha 1. Efficiency of target genes varied from 97.1% to 102% in this study. The relative expression of target genes versus the eef1a1 was calculated according to the formula: R = 2^−ΔΔCT^ [24].

**Table 3:** Effects of dietary starch levels on feed utilization and morphological indices of largemouth bass *M. salmoides*.

Diets	LS	MS	HS
Feed efficiency ratio	1.21 ± 0.01 a	1.25 ± 0.01 ab	1.30 ± 0.04 b
Protein efficiency ratio	2.47 ± 0.01 a	2.54 ± 0.02 ab	2.61 ± 0.08 b
Feeding ratio (%)	2.53 ± 0.01 b	2.43 ± 0.09 ab	2.28 ± 0.12 a
Condition factor (g/cm^3^)	2.85 ± 0.07	2.81 ± 0.01	2.69 ± 0.11
Viscerosomatic index (%)	8.31 ± 0.39 a	9.81 ± 0.78 b	9.85 ± 0.30 b
Hepatosomatic index (%)	2.42 ± 0.10 a	3.52 ± 0.26 ab	4.09 ± 0.77 b
Intraperitoneal fat ratio (%)	1.76 ± 0.23	1.71 ± 0.16	1.75 ± 0.12

Data expressed as mean ± SD (*n* = 3). Different letters in same row indicate significant differences among treatments (*p* < 0.05). LS, low starch; MS, medium starch; HS, high starch.

**Table 4:** Effects of dietary starch levels on blood glucose and serum biochemical indices of largemouth bass *M. salmoides*.

Diets	LS	MS	HS
Glucose (mmol/L)	2.94 ± 0.19	3.04 ± 0.01	2.60 ± 0.28
Triglyceride (mmol/L)	11.1 ± 1.5	13.3 ± 1.3	11.8 ± 0.7
Cholesterol (mmol/L)	10.8 ± 0.6	11.3 ± 1.2	12.0 ± 0.4
Protein (g/L)	24.0 ± 1.9	21.1 ± 0.8	21.9 ± 0.4

Data expressed as mean ± SD (*n* = 3). LS, low starch; MS, medium starch; HS, high starch.

**Table 5:** Effect of dietary starch levels on nutrient composition of largemouth bass *M. salmoides* (wet weight basis).

Diets	LS	MS	HS
Whole body moisture (%)	70.4 ± 0.0	70.3 ± 0.6	71.0 ± 1.0
Whole body protein (%)	17.4 ± 0.6	17.1 ± 0.1	16.9 ± 0.3
Whole body lipid (%)	9.45 ± 0.18	9.11 ± 0.81	8.89 ± 1.07
Whole body ash (%)	3.64 ± 0.03	3.75 ± 0.05	3.75 ± 0.06
Liver moisture (%)	67.1 ± 0.2	66.7 ± 0.4	67.0 ± 0.7
Liver protein (%)	11.2 ± 0.3 b	8.55 ± 0.48 a	7.83 ± 1.05 a
Liver lipid (%)	4.77 ± 0.53	4.88 ± 0.36	4.68 ± 0.14
Liver glycogen (mg/g)	116 ± 10 a	137 ± 8 ab	162 ± 8 b

Data expressed as mean ± SD (*n* = 3). Different letters in same row indicate significant differences among treatments (*p* < 0.05). LS, low starch; MS, medium starch; HS, high starch.

## Data Availability

Raw sequencing data are deposited at NCBI with a bioproject number of PRJNA1158314, and the accession numbers are from SRX26014194 to SRX26014199. The other data are available upon reasonable request.

## Supplementary Materials

The following supporting information can be downloaded at: https://www.mdpi.com/article/10.3390/ani14233394/s1, Here are some files related to the raw data of the experiment: Tables S1 and S2, quality assessment of the transcriptome; Tables S3 and S4, *p* values of target KEGG enriched pathways and DEGs. Figures S1–S5, raw data of target KEGG enriched pathways.

## Abbreviations

Akt, protein kinase B; Bad, Bcl-2-associated death promoter; Bax, Bcl-2-associated X protein; Bcl2, B-cell lymphoma-2; Bid, Bcl-2 homology 3 interacting domain death agonist; caspase 8, cysteine aspartate-specific protease 8; Cd40, cluster of differentiation 40; Col1a1, type I collagen; Ctgf, connective tissue growth factor; Cyt c, cytochrome c; DEGs, differentially expressed genes; diablo, direct IAP binding protein with low pI; ECM, extracellular matrix; Fadd, Fas-associated death domain protein; Fas, factor associated suicide; Fas-l, factor associated suicide ligand; Gli, glioma causative factor; HSCs, hepatic stellate cells; Iap, inhibitors of apoptosis proteins; Iκb, inhibitors of Nfκb; Il-8, interleukin-8; Ip10, interferon-inducible protein 10; Mip-1β, microphage infectivity potentiator 1β; Nfκb, nuclear factor kappa-B; Pi3k, phosphatidylinositol 3 kinase; Ptc, patched homolog; Smo, smoothened homolog; Tgfβ, transforming growth factor β; Tgfβr, transforming growth factor β receptor; Tlr, toll-like receptor; Tnfr, tumor necrosis factor receptor; Trail, Tnf-related apoptosis inducing ligand; Trail-r, Tnf-related apoptosis inducing ligand receptor; Xiap, X-linked apoptosis inhibitory protein.
