# Supplementary material for: Transcriptome Profiling Unveils the Mechanisms of Inflammation, Apoptosis, and Fibrosis in the Liver of Juvenile Largemouth Bass Micropterus salmoides Fed High-Starch Diets
Source: Animals (Basel). 2024 Nov 25;14(23):3394. doi: 10.3390/ani14233394 (PMC11640739; doi:10.3390/ani14233394)

Figure S1 Toll-like signaling pathway from KEGG pathway enrichment

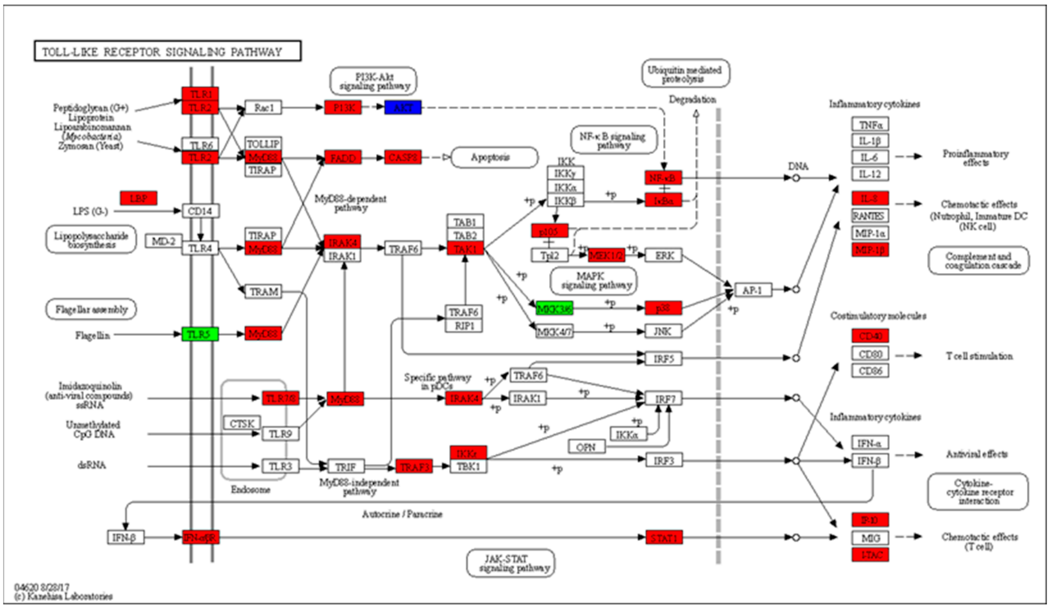

Figure S2 Apoptosis from KEGG pathway enrichment

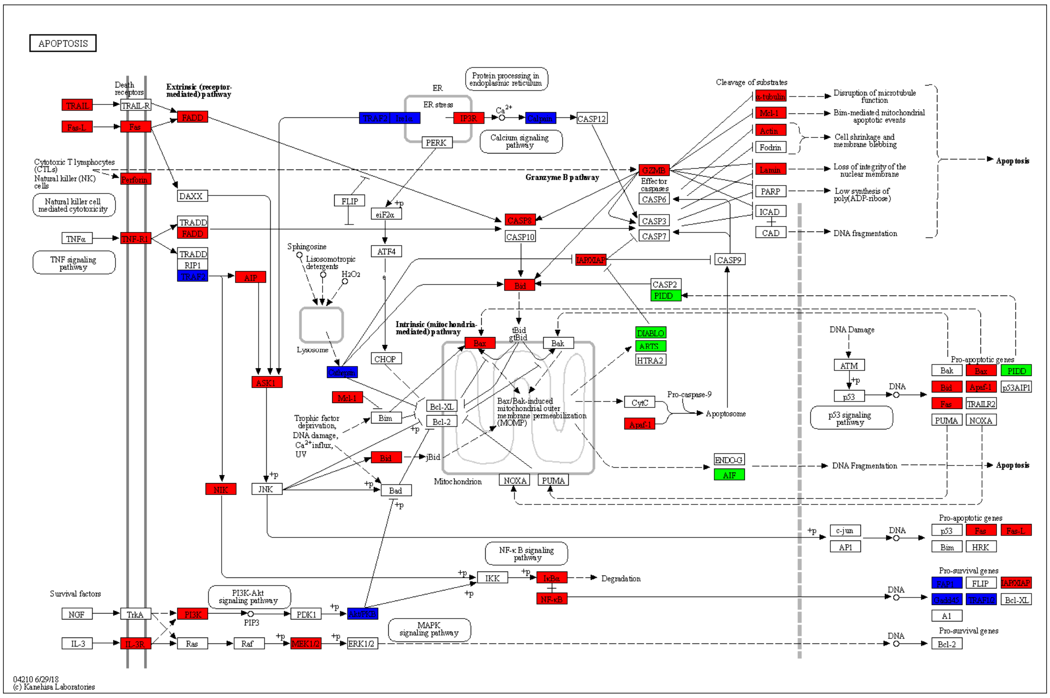

Figure S3 Cytokine-cytokine receptor interaction from KEGG pathway enrichment

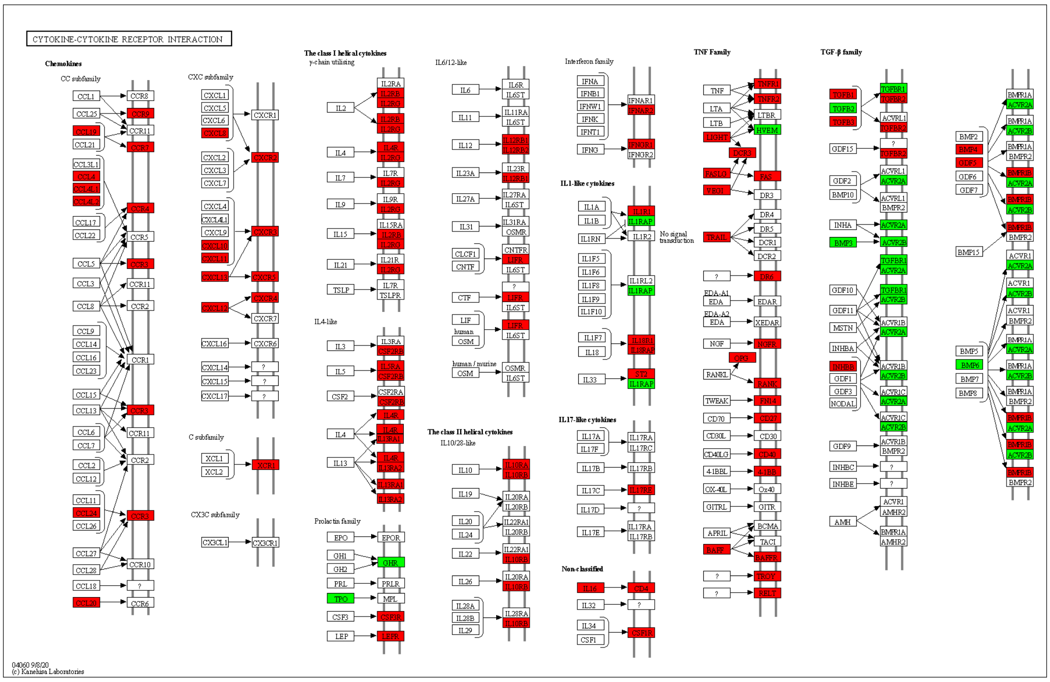

Figure S4 ECM-receptor interaction from KEGG pathway enrichment

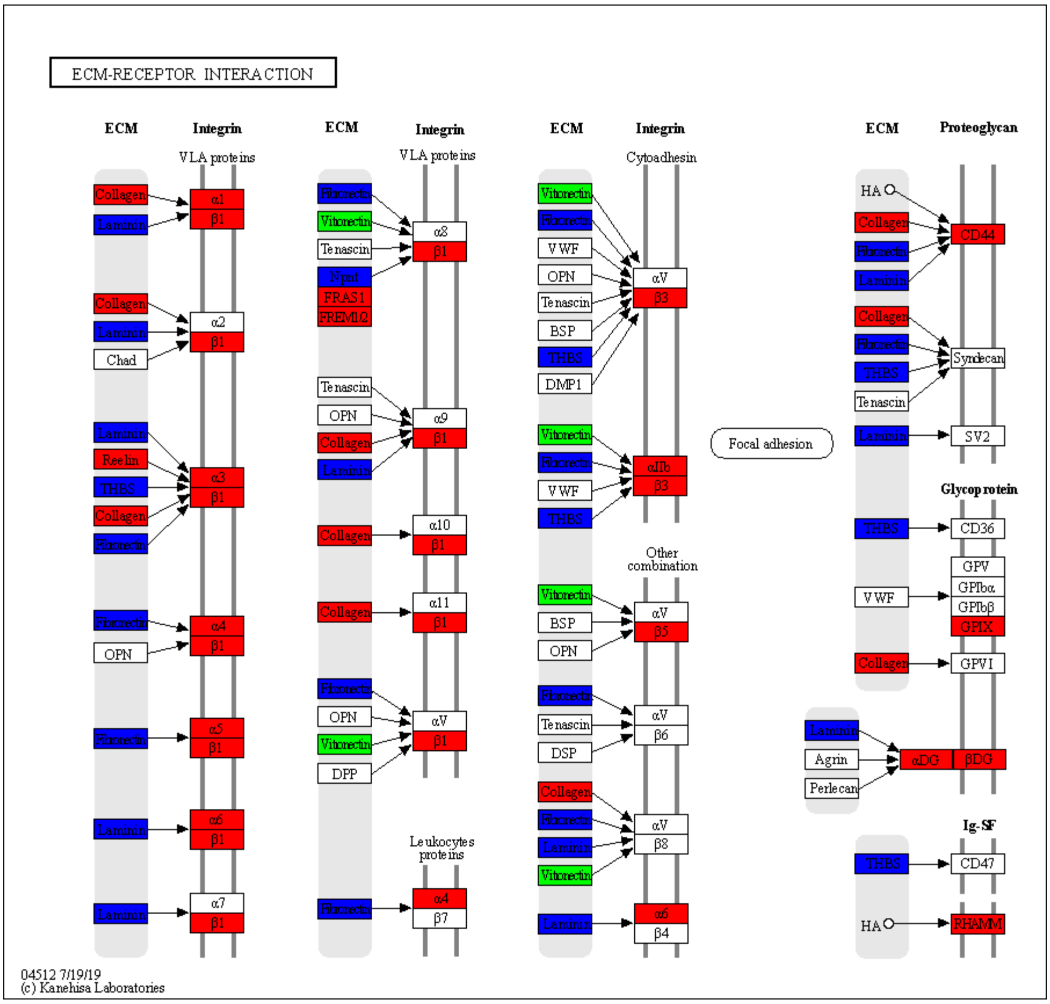

Figure S5 Hedgehog signaling pathway from KEGG pathway enrichment

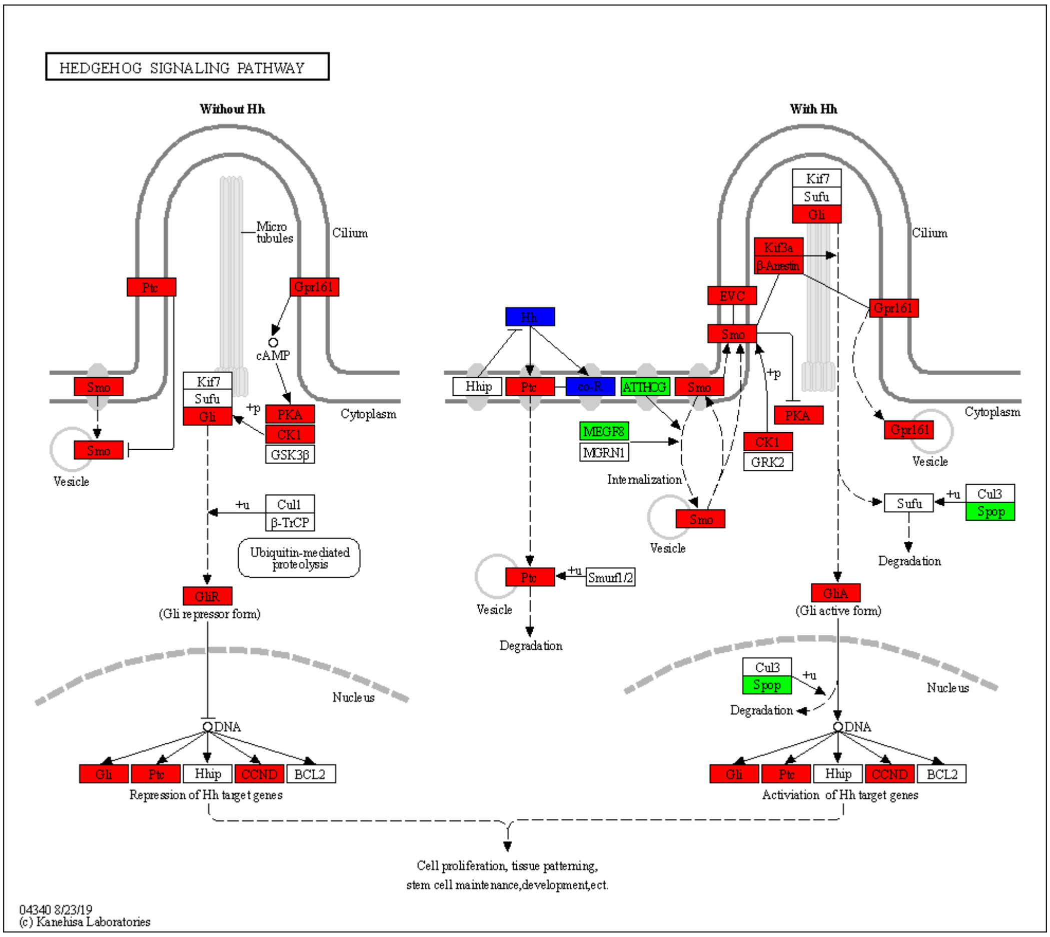

Supplement: Supplementary file 1 [file animals-14-03394-s001.zip › Supplemental Figures.pdf]
