# Supplementary material for: Transcriptome Profiling Unveils the Mechanisms of Inflammation, Apoptosis, and Fibrosis in the Liver of Juvenile Largemouth Bass Micropterus salmoides Fed High-Starch Diets
Source: Animals (Basel). 2024 Nov 25;14(23):3394. doi: 10.3390/ani14233394 (PMC11640739; doi:10.3390/ani14233394)
Supplement: Supplementary file 1 [file animals-14-03394-s001.zip › Supplemental Tables.pdf]

Table S1 Quality assessment of the transcriptome

	Clean reads	Clean bases	GC content	%≥Q30
LS1	19,917,548	5,962,618,932	48.6%	94.5%
LS2	20,245,995	6,060,843,342	48.6%	94.4%
LS3	20,609,776	6,171,088,054	48.3%	94.7%
HS1	19,051,461	5,702,039,404	48.1%	94.7%
HS2	20,696,836	6,193,197,850	47.7%	93.9%
HS3	21,804,782	6,527,036,182	48.1%	94.5%

Table S2 Sequence alignment analysis of the transcriptome with largemouth bass reference genome

	Total Reads	Mapped Reads	Uniq Mapped Reads	Multiple Map Reads
LS1	39,835,096	37,853,587 (95.0%)	35,307,021 (88.6%)	2,546,566 (6.39%)
LS2	40,491,990	38,434,121 (94.9%)	36,043,498 (89.0%)	2,390,623 (5.90%)
LS3	41,219,552	39,130,495 (95.0%)	36,661,734 (88.9%)	2,468,761 (5.99%)
HS1	38,102,922	36,144,752 (94.9%)	34,411,670 (90.3%)	1,733,082 (4.55%)
HS2	41,393,672	38,981,560 (94.2%)	37,063,281 (89.5%)	1,918,279 (4.63%)
HS3	43,609,564	41,113,114 (94.3%)	38,685,621 (88.7%)	2,427,493 (5.57%)

Table S3 Target signaling pathways by KEGG enrichment analysis

ID	Description	Enrich factor	*P* value	No. of differential expressed genes
ko04210	Apoptosis	1.48	< 0.001	70
ko04060	Cytokine-cytokine receptor interaction	1.70	< 0.001	105
ko04512	ECM-receptor interaction	1.73	< 0.001	52
ko04340	Hedgehog signaling pathway	1.46	< 0.05	21
ko04620	Toll-like receptor signaling pathway	1.35	< 0.05	40

Table S4 Feature counts and FDR value of target differentially expressed genes

Accessing no.	Genes	Feature counts						log2 fold change values	FDR value
		LS1	LS2	LS3	HS1	HS2	HS3		
EVM0016028	Tlr1	29	74	25	311	166	729	2.50	< 0.001
EVM0003202	Tlr2	15	29	19	97	63	180	1.72	< 0.001
EVM0004603	Pi3k	104	175	127	384	342	2425	2.14	< 0.01
EVM0002814	Nfkb	507	631	449	1408	922	2600	0.93	< 0.05
EVM0006828	Ikb	726	1002	616	1584	1878	3567	0.90	< 0.05
EVM0001268	Il-8	78	139	93	583	534	7906	3.98	< 0.001
EVM0000450	Ip-10	40	154	64	414	261	650	1.68	< 0.01
EVM0011429	Il3r	86	158	62	740	368	5323	3.55	< 0.001
EVM0021219	Xiap	635	800	643	1799	1325	3266	0.92	< 0.01
EVM0010760	Fas-l	14	26	17	89	66	124	1.62	< 0.001

EVM0000065	Fas	74	75	46	140	160	232	0.80	< 0.05
EVM0013842	Tnfαr	298	525	346	2286	1003	5232	2.11	< 0.001
EVM0013628	Fadd	45	67	50	188	122	318	1.25	< 0.01
EVM0022077	Caspase8	104	129	85	521	220	756	1.53	< 0.01
EVM0003022	Ptc	31	95	33	483	156	1009	2.63	< 0.001
EVM0022965	Smo	196	294	189	748	420	1074	1.03	< 0.01
EVM0019885	Gli	196	294	189	748	420	1074	1.24	< 0.05
EVM0008378	Cyclin 1/2	272	275	176	746	619	658	0.87	< 0.01
EVM0017170	Tgfβ1	267	529	314	1749	793	3689	1.75	< 0.01
EVM0011207	Tgfβ3	471	549	369	2175	1040	3236	1.51	< 0.001
EVM0008262	TgfβrI	564	500	468	539	314	379	-0.94	< 0.01
EVM0005405	TgfβrII	203	240	188	961	457	1808	1.62	< 0.001
EVM0010351	Col1a1	24117	30366	20058	85143	44099	115861	1.03	< 0.01

EVM0024913	Ctgf	187	269	193	854	668	1554	1.55	< 0.001
EVM0018096	Trail	9	8	16	47	34	294	2.66	< 0.01
EVM0016028	Tlr1	29	74	25	311	166	729	2.50	< 0.001
